# Quantification of Liver, Subcutaneous, and Visceral Adipose Tissues by MRI Before and After Bariatric Surgery

**DOI:** 10.1007/s11695-019-03897-2

**Published:** 2019-05-14

**Authors:** Anne Christin Meyer-Gerspach, Ralph Peterli, Michael Moor, Philipp Madörin, Andreas Schötzau, Diana Nabers, Stefan Borgwardt, Christoph Beglinger, Oliver Bieri, Bettina K. Wölnerhanssen

**Affiliations:** 1 St. Clara Research Ltd. at St. Claraspital Basel, CH-4002 Basel, Switzerland; 2 Department of Surgery, St. Claraspital Basel, Basel, Switzerland; 3 Department of Radiology, University of Basel Hospital, Basel, Switzerland; 4 Department of Biomedicine, University of Basel Hospital, Basel, Switzerland; 5 Division of Medical and Biological Informatics, German Cancer Research Center (DKFZ), Heidelberg, Germany; 6 Department of Psychiatry, University of Basel Hospital, Basel, Switzerland

**Keywords:** Obesity, Fatty liver, Adipose tissue, Bariatric surgery, Magnetic resonance imaging

## Abstract

**Background:**

Morbid obesity is a worldwide epidemic and is increasingly treated by bariatric surgery. Fatty liver is a common finding; almost half of all patients with non-alcoholic steatohepatitis develop steatohepatitis. Bariatric surgery improves steatohepatitis documented by liver biopsy and single voxel magnetic resonance imaging (MRI) techniques.

**Objective:**

To investigate changes before and after bariatric surgery using whole organ MRI quantification of liver, visceral, and subcutaneous fat.

**Setting:**

University of Basel Hospital and St. Clara Research Ltd, Basel, Switzerland.

**Methods:**

Sixteen morbidly obese patients were evaluated by abdominal MRI-scanning before and 3, 6, 12, and 24 months after bariatric surgery to measure percentage liver fat (%-LF), total liver volume (TLV) and visceral and subcutaneous adipose tissues (VAT and SAT). Fasting plasma samples were taken for measurement of glucose, insulin, blood lipids, and liver biomarkers. In a control group of 12 healthy lean volunteers, the liver biomarker was also measured.

**Results:**

The reproducibility of fat quantification by use of MRI was excellent. LF decreased significantly faster than VAT and SAT (%-LF vs. VAT *p* < 0.001 and %-LF vs. SAT *p* < 0.001). At certain time points, %-LF, VAT, and SAT were associated with changes in blood lipids and insulin.

**Conclusions:**

MRI quantification offers excellently reproducible results in measurement of liver fat and visceral and subcutaneous adipose tissues. Liver fat decreased significantly faster than visceral or subcutaneous adipose tissue. Decrease in %-LF and VAT is associated with decrease in total cholesterol, LDL, and plasma insulin.

**Electronic supplementary material:**

The online version of this article (10.1007/s11695-019-03897-2) contains supplementary material, which is available to authorized users.

## Introduction

Obesity has become one of the greatest public health challenges in the twenty-first century, with an epidemic prospect that > 50% of the world’s adult population will be overweight and obese by 2030 [1]. Of special interest in this context is fatty liver disease, which is strongly related to BMI and—when progressing to steatohepatitis (NASH) and cirrhosis—is now the second leading etiology for terminal liver failure in the USA [2–4]. Accumulation of fat in non-adipose tissue such as the liver results in lipotoxicity leading to cellular dysfunction and death. The mechanism by which lipotoxicity causes death and dysfunction is not well understood; the extent of cellular dysfunction is related to the type of cell affected, as well as the type and quantity of excess fats [5]. Intrahepatic fat accumulation is associated with increased insulin resistance and promotes the development of type 2 diabetes mellitus (T2DM) [6, 7]. Bariatric surgery is the most effective treatment in morbid obesity (BMI > 35 kg/m^2^) [8]. Weight loss reduces abdominal and intrahepatic fat, thereby improving metabolic and cardiovascular risk [9]. Currently, liver biopsy is still regarded as the gold standard in the assessment of liver fat content, the diagnosis of NAFLD, and monitoring of progression [10]—also because of the big advantage that liver biopsy can differentiate between NAFLD, NASH, cirrhosis, and other histologic changes [11]. However, accuracy of diagnosis is not perfect with a certain risk of sampling error depending on biopsy quality and a more or less homogeneous distribution of liver tissue alterations [12, 13]. Complications are rare; however, the method is invasive and carries a small risk of bleeding. Moreover, morbid obesity is considered to be a relative contraindication for liver biopsy and it cannot be used as a routine method during follow-up period [14]. Therefore, non-invasive methods (such as imaging techniques or biomarkers) are sought for accurate diagnosis and safe monitoring of disease progression.

In recent years, the development of fat–water magnetic resonance imaging (MRI) has enabled non-invasive assessment of fat and water content in tissues. In addition, modern MRI devices allow brief breath holding, which reduces motion artifacts and provides us with excellent data and therefore MRI has become an important tool for fat quantification [15]. Possible biomarkers, which could be helpful in the diagnosis and monitoring of fatty liver diseases, are the following: liver fatty acid-binding proteins (L-FABP), Fetuin A, and M30.

The purpose of this study was to evaluate visceral, subcutaneous, and liver fat distributions in morbidly obese patients before and after bariatric surgery as well as to investigate the dynamic of fat decrease postoperatively. In addition, we aimed to gain basic information on changes in liver biomarkers in morbidly obese patients before and after surgery.

## Materials and Methods

### Subjects

Between April 2014 and November 2016, a total 16 morbidly obese patients (4 scheduled for LSG and 12 for LRYGB; 2 male, 14 female, mean BMI before surgery 40.6 ± 1.0 kg/m^2^, range 35.6–48.9 kg/m^2^, age 34.4 ± 2.9 years, range 19–54 years) and 12 healthy lean controls (12 males; mean age 24.8 years, BMI 22.9 kg/m^2^, range: 21–24) were included in this trial.

### Study Protocol

The study was carried out in accordance with the Declaration of Helsinki. The protocol was submitted and approved by the Local Research and Ethics Committee in Basel. The trial is registered in the Clinical trials registry of the National Institutes of Health (NCT 02682173). Details about inclusion and exclusion criteria are described in the Supplementary Materials and Methods. Morbidly, obese patients took part in a 30-min screening session and 9 trial sessions (3 preoperatively, 3 postoperatively after 3 and after 6, 12, and 24 months). In contrast, healthy lean controls took part in a 30-min screening session and one trial session. On each session, subjects were admitted to the Phase 1 Research Unit, Division of Gastroenterology and Hepatology University Hospital of Basel, Switzerland at 08:00 AM. After an overnight fast of at least 10 h, vital signs were recorded and an antecubital catheter was inserted into a forearm vein for blood collection. In morbidly obese patients, fasting blood samples for measurement of insulin, glucose, and blood lipids were taken and abdominal MR was performed. In addition, in nine morbidly obese patients (0, 3, and 6 months postsurgery) and 12 healthy lean controls, liver biomarkers (L-FABP, Fetuin A, M30) were measured from fasting blood samples.

### Fat Mass Quantification

A Siemens 3T MAGNETOM Prisma scanner at the University Hospital of Basel was used to acquire the abdominal data for evaluation of percentage liver fat (%-LF), total liver volume (TLV), visceral adipose tissue (VAT), and subcutaneous adipose tissue (SAT). In one preoperative session, the abdominal scans were evaluated by a radiologist of the University Hospital of Basel. For the abdominal VAT and SAT quantification, a two-point Dixon (3D multi-echo flash with two echoes) in coronal orientation and for the liver fat measurement, a transversal T2*-IDEAL (3D multi-echo Flash with six echoes) were used. The two-point Dixon multi-echo flash sequence (TA 20 s, TR 4.07 ms, TE 1.23 ms, 2.46 ms, flip angle 9°, FoV 500 × 500 mm, slice thickness 2 mm, 256 × 230 matrix [voxel size 2.0 × 2.0 × 2.0 mm], 192 slices) reached from the diaphragm to the symphysis and acquisition time was 20 s (a parallel acquisition technique with an acceleration factor of 3 and 24 reference lines was used). A T2*-IDEAL six echoes Flash sequence (TA17 s, TR 9.11 ms, TE 1.23 ms, 2.46 ms, 3.69 ms, 4.92 ms, 6.15 ms, 7.32 ms, flip angle 6°, FoV 450 × 337.5 mm, slice thickness 4 mm, 224 × 179 matrix [voxel size 2.0 × 2.0 × 4.0 mm], 56 slices) covered the whole liver in transversal orientation and calculated fat maps, which were used for the liver fat measurement. Both sequences were performed in a single breath hold (in inspiration) and applied in seven sessions (three times before surgery and four times after surgery: at 3, 6, 12, and 24 months).

For the SAT and VAT volume estimations, an automatic segmentation to the two-point Dixon data based on a statistical shape model (SSM) was used [16]. The segmentation results were then manually corrected and spatially confined to the upper end of the femoral head as well as the lower end of the ninth thoracic vertebra using the Medical Imaging Interaction Toolkit (MITK) [17]. T2*-IDEAL images were used to determine the volume of the liver, which was segmented manually using ITK-SNAP [18]. Finally, SAT, VAT, and liver volumes were derived using the voxel count of each segmentation multiplied by the voxel volume of the corresponding imaging sequence.

### Blood Analysis, Data Analysis, Sample Size Estimation, and Statistical Analysis

Details are described in the Supplementary Materials and Methods .

## Results

For BMI, ΔBMI from baseline, excess percentage weight loss (%EWL), %-LF, and TLV, see Table 1. For blood parameters (glucose, insulin, Homeostasis Model Assessment (HOMA) index, total cholesterol, HDL cholesterol, cholesterol/HDL ratio, LDL and triglycerides), see Table 2. Biomarkers L-FABP, Fetuin A, and M30 are presented in Supplementary Results and Supplementary Figure 2.

**Table 1:** Demographic data, percentage liver fat (%-LF), and total liver volume (TLV)

	Before surgery	3 months	6 months	12 months	24 months
BMI (kg/m^2^)	40.6 ± 1.0	33.6 ± 0.9	29.1 ± 0.9	27.5 ± 1.0	27.4 ± 1.5
ΔBMI from baseline (kg/m^2^)	–	− 7.0 ± 0.5	− 10.9 ± 0.9	− 13.4 ± 1.2	− 13.5 ± 1.5
%EWL	–	46.4 ± 3.6	73.7 ± 5.5	88.6 ± 6.5	86.7 ± 9.3
%-LF	13.0 ± 2.4	6.0 ± 1.7	3.9 ± 1.7	1.5 ± 0.3	2.3 ± 0.6
Decline in LF vs. before surgery (%)	–	64.2 ± 4.7	74.8 ± 5.2	80.3 ± 4.5	79.2 ± 7.2
TLV (cm^3^)	1725.5 ± 92.1	1348.6 ± 71.2	1266.5 ± 51.4	1426.1 ± 65.4	1424.6 ± 85.9

Data are reported as mean ± SEM

*BMI* body mass index, *%-LF* percentage liver fat, *TLV* total liver volume, *%EWL* weight loss defined by excess percentage weight loss

**Table 2:** Blood parameters

	Before surgery	3 months	6 months	12 months	24 months	*p* values
Total cholesterol (mmol/L)	4.6 ± 0.2	–	3.9 ± 0.2	4.0 ± 0.2	4.4 ± 0.2	*p* = 0.001^a^ *p* = 0.023^c^ *p* = 1.000^d^
HDL cholesterol (mmol/L)	1.3 ± 0.1	–	1.4 ± 0.1	1.5 ± 0.1	2.0 ± 0.1	*p* = 0.965^a^ *p* = 0.097^c^ *p* < 0.001^d^
Cholesterol/HDL ratio (mmol/L)	3.7 ± 0.3	–	2.9 ± 0.1	2.7 ± 0.2	2.4 ± 0.1	*p* = 0.027^a^ *p* = 0.001^c^ *p* < 0.001^d^
LDL(mmol/L)	2.9 ± 0.2	–	2.2 ± 0.2	2.1 ± 0.2	2.3 ± 0.2	*p* = 0.001^a^ *p* < 0.001^c^ *p* = 0.152^d^
Triglycerides (mmol/L)	1.5 ± 0.3	–	0.9 ± 0.1	0.8 ± 0.1	1.1 ± 0.1	*p* = 0.113^a^ *p* = 0.091^c^ *p* = 0.850^d^
Fasting glucose (mmol/L)	5.3 ± 0.2	4.7 ± 0.1	4.6 ± 0.1	4.7 ± 0.1	–	*p* = 0.002^a^ *p* = 0.001^b^ *p* = 0.006^c^
Fasting insulin (μU/mL)	7.3 ± 1.0	2.9 ± 0.3	2.8 ± 0.7	2.5 ± 0.4	–	*p* = 0.001^a^ *p* = 0.007^b^ *p* = 0.003^c^
HOMA index	1.7 ± 0.2	0.6 ± 0.1	0.6 ± 0.1	0.5 ± 0.1	–	*p* < 0.001^a^ *p* = 0.004^b^ *p* = 0.002^c^

Blood parameters are reported as mean ± SEM. Statistics: ANOVA multiple comparisons with Bonferroni correction. Homeostasis Model Assessment index—a measure for insulin resistance—was calculated based on the formula: HOMA IR = (fasting glucose (mmol/L) × fasting insulin (μU/mL))/22.5

^a^Before surgery vs. 3 months

^b^Before surgery vs. 6 months

^c^Before surgery vs. 12 months

^d^Before surgery vs. 24 months

### Fat Mass Quantification

#### Reproducibility

The reproducibility of fat quantification (measured at the first three presurgical visits) was estimated by intraclass correlation (ICC) of baseline fat values. The ICC ranges between 0 and 1, where 1 means perfect agreement, and a value between 0.75 and 1.00 can be marked as excellent. The ICC for %-LF was 0.88, TLV 0.83, VAT 0.94, and SAT 0.90.

#### Time Courses and Comparison of Exponential Decay Constants Between TLV, %-LF, SAT, and VAT

**Fig. 1:**
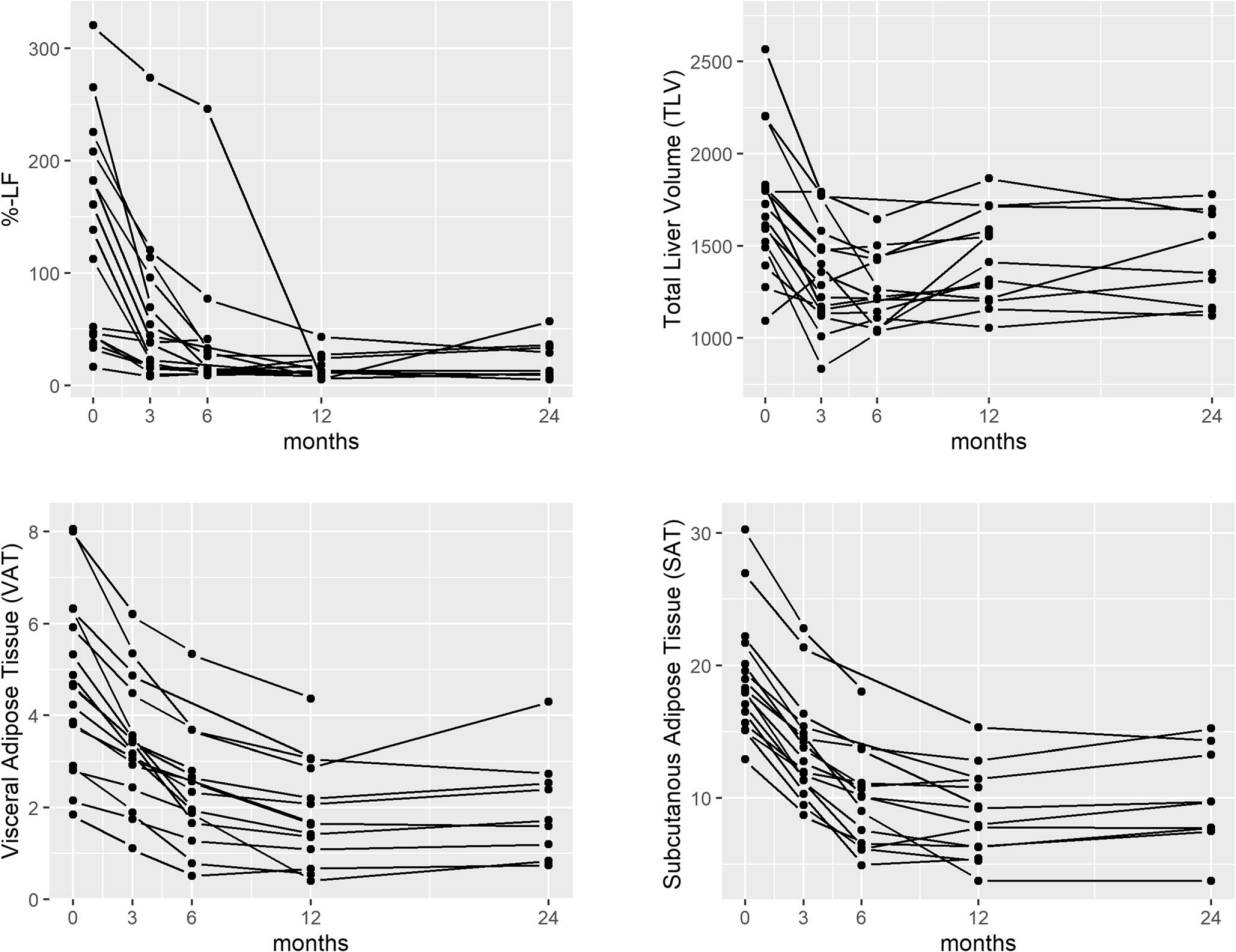
Time courses of percentage liver fat (%-LF), total liver volume (TLV), visceral adipose tissue (VAT), and subcutaneous adipose tissue (SAT). Time courses of %-LF, TLV, VAT, and SAT before and 3, 6, 12, and 24 months after surgery. *N* = 16 morbidly obese patients. %-LF percentage liver fat, TLV total liver volume, VAT visceral adipose tissue, SAT subcutaneous adipose tissue

TLV, %-LF, SAT, and VAT decreased after surgery (Fig. 1; Table 1). Time courses of all parameters followed the proposed exponential model with good agreement. Results are reported as A = log(initial value), B = decay, and A1 = slope of ascending process (Table 3). For all parameters, the ascending slope at the end was statistically significant, indicating an increase of the respective parameters after a certain nadir has been reached. %-LF decreased significantly faster than VAT and SAT (%-LF vs. VAT *p* < 0.001 and %-LF vs. SAT *p* < 0.001), while there was no difference when comparing the exponential decay constants between VAT and SAT (SAT vs. VAT: *p* = 0.94; Table 4; Fig. 1).

**Table 3:** Time courses of HOMA and fat values

	Non-linear regression coefficient ± SE, (*p* value)		
	A = (log)initial value	B = decay	A1 = slope of ascending process
HOMA	0.4470 ± 0.1346	− 0.3460 ± 0.0596	0.0402 ± 0.0066
	(0.0019)	(< 0.001)	(< 0.001)
%-LF	2.3328 ± 0.2156	− 0.2084 ± 0.0336	0.0417 ± 0.0182
	(< 0.001)	(< 0.001)	(0.0238)
TLV	7.4392 ± 0.0505	− 0.0839 ± 0.0088	48.5448 ± 3.5344
	(< 0.001)	(< 0.001)	(< 0.001)
VAT volume	1.5025 ± 0.1050	− 0.0989 ± 0.0083	0.0492 ± 0.0106
	(< 0.001)	(< 0.001)	(< 0.001)
SAT volume	2.9518 ± 0.0540	− 0.0999 ± 0.0065	0.2787 ± 0.0368
	(< 0.001)	(< 0.001)	(< 0.001)

Non-linear regression coefficient is reported as mean ± SE. Statistic: nonlinear mixed-effects models

*%-LF* percentage liver fat, *TLV* total liver volume, *VAT* visceral adipose tissue, *SAT* subcutaneous adipose tissue

**Table 4:** Comparison of exponential decay constants between fat values

	A: %-LF	B: TLV	C: VAT volume	D: SAT volume
Exponential decay constant (SD)	0.21 ± 0.09	0.08 ± 0.02	0.10 ± 0.03	0.10 ± 0.02
	B: 0.0012			
	C: < 0.001	C: 0.231		
	D: < 0.001	D: 0.058	D: 0.94	

Exponential decay is reported as mean ± SD. Statistic: paired Wilcoxon tests

*%-LF* percentage liver fat, *TLV* total liver volume, *VAT* visceral adipose tissue, *SAT* subcutaneous adipose tissue

### Associations Between %-LF and Blood Parameters

#### %-LF vs. Plasma Insulin, Plasma Glucose, and HOMA

While %-LF was not associated with fasting glucose at any time point, there was an association with fasting insulin (*p* = 0.002) and subsequently HOMA (*p* = 0.003) before surgery. Furthermore, Δ%-LF vs. Δfasting insulin showed an association at 12 months after surgery (*p* = 0.003) and so did HOMA (*p* = 0.004); Table 5.

**Table 5:** Associations between percentage liver fat (%-LF) and blood parameters

Correlation	Regression coefficient ± SE (*p* value)				
	Before surgery	3 months	6 months	12 months	24 months
A: %-LF vs. blood parameter					
Total cholesterol	0.066 ± 0.021 (0.007)	–	0.068 ± 0.032 (0.059)	− 0.220 ± 0.336 (0.534)	0.117 ± 0.164 (0.516)
HDL cholesterol	− 0.004 ± 0.009 (0.687)	–	− 0.005 ± 0.017 (0.773)	0.039 ± 0.083 (0.654)	0.010 ± 0.123 (0.942)
Cholesterol/HDL ratio	0.058 ± 0.028 (0.057)	–	0.056 ± 0.030 (0.091)	− 0.242 ± 0.251 (0.368)	0.012 ± 0.121 (0.925)
LDL	0.070 ± 0.018 (0.002)	–	0.079 ± 0.032 (0.035)	− 0.235 ± 0.317 (0.483)	0.013 ± 0.153 (0.935)
Triglycerides	0.027 ± 0.029 (0.367)	–	0.013 ± 0.017 (0.462)	− 0.081 ± 0.096 (0.427)	0.205 ± 0.068 (0.040)
Fasting glucose	0.004 ± 0.015 (0.793)	− 0.006 ± 0.015 (0.690)	− 0.011 ± 0.015 (0.490)	− 0.103 ± 0.095 (0.303)	–
Fasting insulin	0.299 ± 0.077 (0.002)	0.067 ± 0.042 (0.137)	− 0.051 ± 0.114 (0.662)	− 0.089 ± 0.376 (0.816)	–
HOMA index	0.073 ± 0.020 (0.003)	0.013 ± 0.010 (0.192)	− 0.011 ± 0.022 (0.635)	− 0.035 ± 0.084 (0.681)	–
B: %-LF difference from preoperative value vs. blood parameter difference from preoperative value					
Total cholesterol	–	–	0.027 ± 0.015 (0.106)	0.007 ± 0.016 (0.684)	0.044 ± 0.019 (0.083)
HDL cholesterol	–	–	− 0.003 ± 0.011 (0.791)	− 0.006 ± 0.007 (0.426)	− 0.005 ± 0.010 (0.655)
Cholesterol/HDL ratio	–	–	0.031 ± 0.042 (0.485)	0.017 ± 0.018\ (0.369)	0.048 ± 0.029 (0.171)
LDL	–	–	0.024 ± 0.013 (0.091)	0.022 ± 0.012\ (0.114)	0.058 ± 0.019\ (0.036)
Triglycerides	–	–	0.022 ± 0.041 (0.612)	− 0.004 ± 0.017 (0.823)	− 0.006 ± 0.012 (0.655)
Fasting glucose	–	0.006 ± 0.019 (0.754)	0.010 ± 0.019 (0.615)	0.013 ± 0.014 (0.357)	–
Fasting insulin	–	0.148 ± 0.153 (0.351)	0.159 ± 0.160 (0.340)	0.337 ± 0.091 (0.003)	–
HOMA index	–	0.034 ± 0.040 (0.417)	0.037 ± 0.040(0.371)	0.082 ± 0.023 (0.004)	–

Regression coefficients are reported as mean ± SE. Statistic: linear regression models. A: describes absolute values: %-LF vs. blood parameters; B: describes the difference in %-LF from preoperative value vs. the difference from preoperative value of each blood parameter

*%-LF* percentage liver fat

#### %-LF vs. Blood Lipids

While %-LF showed an association with total cholesterol before surgery (*p* = 0.007), only a trend was seen after 6 months (*p* = 0.059) and no association was found at 12 and 24 months after surgery. No association between %-LF and HDL values was seen at any time point. The cholesterol/HDL ratio showed a trend before surgery (*p* = 0.057). %-LF showed an association with LDL before surgery (*p* = 0.002) and after 6 months (*p* = 0.035) and with triglycerides only at 24 months after surgery (*p* = 0.04). Taking the difference to the preoperative value in %-LF vs. difference to the preoperative value in blood lipids into account, Δ%-LF vs. ΔLDL showed an association at 24 months after surgery (*p* = 0.036); Table 5.

### Associations Between VAT and Blood Parameters

#### VAT vs. Plasma Insulin, Plasma Glucose, and HOMA

While VAT was not associated with fasting glucose at any time point, there was an association with fasting insulin (*p* = 0.014) and subsequently HOMA (*p* = 0.012) before surgery. Furthermore, ΔVAT vs. Δfasting insulin showed an association at 3 and 6 months after surgery (*p* = 0.005 and *p* = 0.021) and so did HOMA (*p* = 0.006 and *p* = 0.019); Table 6.

**Table 6:** Associations between visceral adipose tissue (VAT) and blood parameters

Correlation	Regression coefficient ± SE (*p* value)				
	Before surgery	3 months	6 months	12 months	24 months
A: VAT vs. blood parameter					
Total cholesterol	0.177 ± 0.130 (0.196)	–	0.079 ± 0.192 (0.690)	0.118 ± 0.320 (0.724)	0.448 ± 0.139 (0.032)
HDL cholesterol	− 0.030 ± 0.046 (0.533)	–	− 0.039 ± 0.092 (0.684)	− 0.051 ± 0.077 (0.529)	0.227 ± 0.149 (0.202)
Cholesterol/HDL ratio	0.201 ± 0.154 (0.214)	–	0.154 ± 0.184 (0.423)	0.177 ± 0.240 (0.486)	− 0.053 ± 0.182 (0.784)
LDL	0.156 ± 0.125 (0.232)	–	0.183 ± 0.216 (0.419)	0.147 ± 0.302 (0.642)	0.090 ± 0.227 (0.713)
Triglycerides	0.169 ± 0.146 (0.267)	–	0.028 ± 0.095 (0.774)	0.068 ± 0.090 (0.478)	0.301 ± 0.110 (0.053)
Fasting glucose	0.067 ± 0.074 (0.378)	0.122 ± 0.072 (0.112)	0.052 ± 0.075 (0.503)	0.144 ± 0.079 (0.093)	–
Fasting insulin	1.259 ± 0.449 (0.014)	0.355 ± 0.215 (0.120)	0.418 ± 0.553 (0.464)	− 0.070 ± 0.336 (0.838)	–
HOMA index	0.323 ± 0.111 (0.012)	0.092 ± 0.045 (0.062)	0.096 ± 0.108 (0.391)	0.010 ± 0.076 (0.900)	–
B: VAT difference from preoperative value vs. blood parameter difference from preoperative value					
Total cholesterol	–	–	0.092 ± 0.138 (0.518)	0.166 ± 0.123 (0.218)	0.031 ± 0.305 (0.923)
HDL cholesterol	–	–	0.074 ± 0.086 (0.416)	0.022 ± 0.058 (0.720)	− 0.079 ± 0.106 (0.501)
Cholesterol/HDL ratio	–	–	− 0.095 ± 0.336 (0.783)	0.060 ± 0.158 (0.713)	0.128 ± 0.392 (0.760)
LDL	–	–	− 0.024 ± 0.119 (0.844)	0.051 ± 0.123 (0.691)	0.141 ± 0.354 (0.711)
Triglycerides	–	–	0.040 ± 0.328 (0.905)	0.279 ± 0.095 (0.022)	0.068 ± 0.128 (0.622)
Fasting glucose	–	0.045 ± 0.155 (0.777)	0.150 ± 0.124 (0.250)	0.092 ± 0.105 (0.398)	–
Fasting insulin	–	3.121 ± 0.945 (0.005)	2.386 ± 0.898 (0.021)	1.458 ± 0.915 (0.137)	–
HOMA index	–	0.805 ± 0.250 (0.006)	0.604 ± 0.223 (0.019)	0.386 ± 0.222 (0.108)	–

Regression coefficients are reported as mean ± SE. Statistic: linear regression models. A: describes absolute values: VAT vs. blood parameters; B: describes the difference in VAT from preoperative value vs. the difference from preoperative value of each blood parameter

*VAT* visceral adipose tissue

#### VAT vs. Blood Lipids

While VAT showed no association with total cholesterol before surgery and 6 and 12 months after surgery, there was an association at 24 at months after surgery (*p* = 0.032). No association between VAT and HDL and LDL and cholesterol/HDL ratio was seen at any time point. VAT showed a trend with triglycerides only at 24 months after surgery (*p* = 0.053). Taking the difference to the preoperative value in ΔVAT vs. difference to the preoperative value in blood lipids into account, ΔVAT vs. Δtriglycerides showed an association at 12 months after surgery (*p* = 0.022); Table 6.

### Associations Between SAT and Blood Parameters

#### SAT vs. Plasma Insulin, Plasma Glucose, and HOMA

SAT was not associated with fasting glucose, insulin, and HOMA at any time point before surgery, but there was a trend for fasting glucose at 12 months after surgery (*p* = 0.055). While ΔSAT vs. Δfasting insulin and HOMA showed no association at any time point, ΔSAT vs. Δfasting glucose showed an association at 12 months after surgery (*p* = 0.046); Table 7.

**Table 7:** Associations between subcutaneous adipose tissue (SAT) and blood parameters

Correlation	Regression coefficient ± SE (*p* value)				
	Before surgery	3 months	6 months	12 months	24 months
A: SAT vs. blood parameter					
Total cholesterol	0.009 ± 0.058 (0.881)	–	0.022 ± 0.067 (0.752)	− 0.034 ± 0.144 (0.822)	0.044 ± 0.090 (0.653)
HDL cholesterol	0.038 ± 0.017 (0.041)	–	0.023 ± 0.030 (0.465)	0.010 ± 0.035 (0.790)	0.090 ± 0.048 (0.135)
Cholesterol/HDL ratio	− 0.093 ± 0.064 (0.168)	–	0.000 ± 0.064 (0.998)	− 0.010 ± 0.111 (0.929)	− 0.057 ± 0.058 (0.383)
LDL	− 0.018 ± 0.055 (0.751)	–	0.014 ± 0.075 (0.855)	− 0.037 ± 0.137 (0.793)	− 0.042 ± 0.078 (0.618)
Triglycerides	− 0.039 ± 0.063 (0.548)	–	− 0.013 ± 0.032 (0.700)	− 0.002 ± 0.042 (0.971)	0.003 ± 0.065 (0.963)
Fasting glucose	0.004 ± 0.0032 (0.908)	− 0.021 ± 0.026 (0.429)	− 0.001 ± 0.027 (0.968)	0.056 ± 0.026 (0.055)	–
Fasting insulin	− 0.122 ± 0.233 (0.608)	0.038 ± 0.080 (0.641)	0.088 ± 0.198 (0.663)	− 0.093 ± 0.113 (0.425)	–
HOMA index	− 0.024 ± 0.059 (0.695)	0.005 ± 0.018 (0.783)	0.019 ± 0.039 (0.639)	− 0.013 ± 0.026 (0.629)	–
B: SAT difference from preoperative value vs. blood parameter difference from preoperative value					
Total cholesterol	–	–	− 0.059 ± 0.044 (0.208)	− 0.001 ± 0.044 (0.977)	0.004 ± 0.067 (0.954)
HDL cholesterol	–	–	0.049 ± 0.027 (0.098)	0.006 ± 0.018 (0.772)	0.017 ± 0.023 (0.501)
Cholesterol/HDL ratio	–	–	− 0.161 ± 0.105 (0.158)	− 0.019 ± 0.050 (0.715)	− 0.016 ± 0.087 (0.863)
LDL	–	–	− 0.040 ± 0.039 (0.339)	− 0.006 ± 0.040 (0.887)	− 0.017 ± 0.079 (0.838)
Triglycerides	–	–	− 0.089 ± 0.110 (0.439)	0.041 ± 0.042 (0.370)	0.031 ± 0.025 (0.283)
Fasting glucose	–	0.109 ± 0.082 (0.202)	0.040 ± 0.053 (0.461)	0.073 ± 0.033 (0.046)	–
Fasting insulin	–	− 0.279 ± 0.700 (0.697)	− 0.220 ± 0.463 (0.643)	− 0.035 ± 0.362 (0.923)	–
HOMA index	–	− 0.047 ± 0.184 (0.803)	− 0.045 ± 0.116 (0.705)	0.014 ± 0.089 (0.879)	–

Regression coefficients are reported as mean ± SE. Statistic: linear regression models. A: describes absolute values: SAT vs. blood parameters; B: describes the difference in SAT from preoperative value vs. the difference from preoperative value of each blood parameter

*SAT* subcutaneous adipose tissue

#### SAT vs. Blood Lipids

While SAT showed an association with HDL before surgery (*p* = 0.041), no association between SAT and total cholesterol, cholesterol/HDL ratio, and LDL and triglycerides was seen at any time point. Taking the difference to the preoperative value in SAT vs. difference to the preoperative value in blood lipids into account, there was no association for any blood lipid parameter at any time point; Table 7.

## Conclusion

The main findings of this trial are the following: (i) the reproducibility of fat quantification by use of MRI was excellent for percentage liver fat (%-LF), total liver volume (TLV), visceral adipose tissue (VAT), and subcutaneous adipose tissue (SAT); (ii) %-LF decreased significantly faster than VAT and SAT—no difference was observed between VAT and SAT; (iii) before surgery, %-LF was associated with plasma insulin, total cholesterol, and LDL; VAT was associated with plasma insulin; and SAT was associated with HDL; (iv) L-FABP was significantly higher in morbidly obese vs. lean controls, while Fetuin A and M30 were not statistically different, and (v) L-FABP decreased postoperatively (statistically significant), M30 decreased (numerically only), while Fetuin A concentrations did not change.

Since there is a strong relationship between obesity and NAFLD, it is of clinical interest to assess the liver fat fraction in obese individuals before and after bariatric surgery. Not all obese patients have the same metabolic risk: Some obese patients develop obesity-related comorbidities even with a relatively low BMI in contrast to others with a significantly higher BMI; thus, it seems that apart from liver fat fraction, the distribution of body fat is essential [19, 20]. Visceral adipose tissue (VAT) is associated with the highest risk to develop comorbidities [21, 22]. However, the loss of body fat observed after bariatric surgery does not automatically lead to a decrease in VAT [20]. Consequently, not only the quantification but also the assessment of the individual distribution of body fat prior to a bariatric surgery as well as during the postoperative course is of high importance. In our trial, a clear decrease in liver fat, VAT, and SAT after bariatric surgery was observed. The postoperative decrease is in line with previous findings in obese patients who underwent RYGB [20, 23]. Furthermore, Luo et al. could demonstrate that 83.7% of the examined bariatric patients with bio-optically tested NAFLD had resolution of hepatosteatosis (under 5%-LF) 6 months after surgery [24]. The present study extents these findings by showing that the decrease of body fat after bariatric surgery follows a distinct time pattern: %-LF decreased significantly faster than VAT and SAT. From these findings, we speculate that liver fat, SAT, and VAT are differently regulated. The decrease in %-LF is associated with decrease in blood lipids (total cholesterol and LDL) and plasma insulin after surgery, and the decrease in VAT is associated with changes in insulin, indicating a clear improvement in obesity-associated metabolic risk factors. The decrease in SAT, on the other hand, was not associated with any clear improvements in blood lipids or glycemic control, confirming the particular role of liver fat and visceral fat in metabolic disease.

Besides liver biopsies, imaging techniques like ultrasound, unidimensional transient elastography (TE; FibroScan®), computed tomography (CT), and magnetic resonance imaging (MRI) are also used in the evaluation of liver diseases [11]. Unidimensional transient elastography is a technique, which can be a helpful tool: however, accuracy of diagnosis is depending on the stage of fibrosis and lower grades of fibrosis (stages 1 and 2) are difficult to assess [25]. Moreover, in obese patients, this method cannot be applied [26, 27]. While MRI and CT easily provide a complete coverage of the liver, abdominal ultrasound has some limitations in particular in morbidly obese patients [28]. MRI combines a good reproducibility with a valid detection of disease progression [11, 29, 30]. Up to date, only a few studies have focused on MR-fat quantification before and after bariatric surgery: patients treated with laparoscopic gastric banding (a purely restrictive procedure) and patients undergoing bariatric surgery (such as laparoscopic sleeve gastrectomy and laparoscopic gastric bypass) have been examined pre- and postoperatively by means of MRI-fat quantification [20, 31, 32]. However, in these studies for liver fat quantification, single voxel magnetic resonance spectroscopy was used, while we assessed full organ volume, which provided us with more representative data. In the present study, the reproducibility of fat quantification by use of MRI was excellent for %-LF, TLV, VAT, and SAT, supporting MRI as an important tool for fat quantification. Our findings are in line with a recent prospective trial showing that the MRI technique is clinically feasible for monitoring liver fat over time [15].

Besides the described imaging techniques, liver biomarkers are in discussion to be helpful in the diagnosis and monitoring of fatty liver diseases. In this trial, Fetuin A concentrations did not change 6 months postsurgery, which is in line with other previous trials studying short-term effects of bariatric surgery on Fetuin A [33]. In contrast, in the long-term (> 12 months), Fetuin A seems to decrease as shown by other groups [34, 35]. In our trial, sample size for evaluation of plasma L-FABP and M30 was probably too small, and although numerically, a decrease after bariatric surgery could be shown, no statistical significance was reached.

Some limitations of our study require consideration. First, the fasting insulin levels (and therefore also HOMA-IR) was surprisingly low in the morbidly obese patients, suggesting that the patients examined were unusually insulin sensitive. Second, in the MRI-fat quantification study, the majority of patients were female (14 out of 16) as in most bariatric cohorts and most of them had gastric bypass surgery (12 out of 16). On the other hand, biomarker studies were carried out in a control group of male, lean volunteers. Our results might therefore not generalize to both sexes.

In conclusion, MRI quantification offers excellently reproducible results in measurement of liver fat, visceral and subcutaneous adipose tissue. Liver fat decreased significantly faster than visceral or subcutaneous adipose tissue. Plasma LDL and insulin concentration are associated with %-LF and visceral fat at certain time points. The biomarkers Fetuin A and M30 yielded no clear results, while L-FABP clearly decreased.

## Electronic Supplementary Material

ESM 1 (DOCX 48 kb)

ESM 2 (PNG 201 kb)

High resolution image (TIF 76 kb)

## References

[1] Kelly T, Yang W, Chen CS, Reynolds K, He J (2008). Global burden of obesity in 2005 and projections to 2030. Int J Obes.

[2] Starley BQ, Calcagno CJ, Harrison SA (2010). Nonalcoholic fatty liver disease and hepatocellular carcinoma: a weighty connection. Hepatology.

[3] Katrina Loomis A et al. Body mass index and risk of non-alcoholic fatty liver disease: two electronic health record prospective studies. J Clin Endocrinol Metab. 2015:jc20153444. 10.1210/jc.2015-3444 PMC4803162 26672639

[4] Wong RJ, Aguilar M, Cheung R, Perumpail RB, Harrison SA, Younossi ZM, Ahmed A (2015). Nonalcoholic steatohepatitis is the second leading etiology of liver disease among adults awaiting liver transplantation in the United States. Gastroenterology.

[5] Gonzalez-Cantero J, Martin-Rodriguez JL, Gonzalez-Cantero A, Arrebola JP, Gonzalez-Calvin JL (2018). Insulin resistance in lean and overweight non-diabetic Caucasian adults: study of its relationship with liver triglyceride content, waist circumference and BMI. PLoS One.

[6] Korenblat KM, Fabbrini E, Mohammed BS, Klein S (2008). Liver, muscle, and adipose tissue insulin action is directly related to intrahepatic triglyceride content in obese subjects. Gastroenterology.

[7] Stefan N, Kantartzis K, Haring HU (2008). Causes and metabolic consequences of fatty liver. Endocr Rev.

[8] Sjostrom L (2007). Effects of bariatric surgery on mortality in Swedish obese subjects. N Engl J Med.

[9] Haufe S, Haas V, Utz W, Birkenfeld AL, Jeran S, Bohnke J, Mahler A, Luft FC, Schulz-Menger J, Boschmann M, Jordan J, Engeli S (2013). Long-lasting improvements in liver fat and metabolism despite body weight regain after dietary weight loss. Diabetes Care.

[10] Joseph AE (1991). Comparison of liver histology with ultrasonography in assessing diffuse parenchymal liver disease. Clin Radiol.

[11] Schwenzer NF, Springer F, Schraml C, Stefan N, Machann J, Schick F (2009). Non-invasive assessment and quantification of liver steatosis by ultrasound, computed tomography and magnetic resonance. J Hepatol.

[12] Mehta SH, Lau B, Afdhal NH, Thomas DL (2009). Exceeding the limits of liver histology markers. J Hepatol.

[13] Martinez SM (2011). Noninvasive assessment of liver fibrosis. Hepatology.

[14] Rockey DC, Caldwell SH, Goodman ZD, Nelson RC, Smith AD (2009). Liver biopsy. Hepatology.

[15] Pooler BD et al. Monitoring fatty liver disease with MRI following bariatric surgery: a prospective, dual-center study. Radiology. 2018:181134. 10.1148/radiol.2018181134 PMC6394737 30561273

[16] Wald D, Teucher B, Dinkel J, Kaaks R, Delorme S, Boeing H, Seidensaal K, Meinzer HP, Heimann T (2012). Automatic quantification of subcutaneous and visceral adipose tissue from whole-body magnetic resonance images suitable for large cohort studies. J Magn Reson Imaging.

[17] Wolf I, Vetter M, Wegner I, Böttger T, Nolden M, Schöbinger M, Hastenteufel M, Kunert T, Meinzer HP (2005). The medical imaging interaction toolkit. Med Image Anal.

[18] Yushkevich PA, Piven J, Hazlett HC, Smith RG, Ho S, Gee JC, Gerig G (2006). User-guided 3D active contour segmentation of anatomical structures: significantly improved efficiency and reliability. Neuroimage.

[19] Wildman RP (2008). The obese without cardiometabolic risk factor clustering and the normal weight with cardiometabolic risk factor clustering: prevalence and correlates of 2 phenotypes among the US population (NHANES 1999-2004). Arch Intern Med.

[20] Otto M, Färber J, Haneder S, Michaely H, Kienle P, Hasenberg T (2015). Postoperative changes in body composition--comparison of bioelectrical impedance analysis and magnetic resonance imaging in bariatric patients. Obes Surg.

[21] Fox CS, Massaro JM, Hoffmann U, Pou KM, Maurovich-Horvat P, Liu CY, Vasan RS, Murabito JM, Meigs JB, Cupples LA, D’Agostino RB, O’Donnell CJ (2007). Abdominal visceral and subcutaneous adipose tissue compartments: association with metabolic risk factors in the Framingham Heart Study. Circulation.

[22] Kaess BM, Pedley A, Massaro JM, Murabito J, Hoffmann U, Fox CS (2012). The ratio of visceral to subcutaneous fat, a metric of body fat distribution, is a unique correlate of cardiometabolic risk. Diabetologia.

[23] Hedderich DM, Hasenberg T, Haneder S, Schoenberg SO, Kücükoglu Ö, Canbay A, Otto M (2017). Effects of bariatric surgery on non-alcoholic fatty liver disease: magnetic resonance imaging is an effective, non-invasive method to evaluate changes in the liver fat fraction. Obes Surg.

[24] Luo RB, Suzuki T, Hooker JC, Covarrubias Y, Schlein A, Liu S, Schwimmer JB, Reeder SB, Funk LM, Greenberg JA, Campos GM, Sandler BJ, Horgan S, Sirlin CB, Jacobsen GR (2018). How bariatric surgery affects liver volume and fat density in NAFLD patients. Surg Endosc.

[25] Friedrich-Rust M (2008). Performance of transient elastography for the staging of liver fibrosis: a meta-analysis. Gastroenterology.

[26] Stevenson M, Lloyd-Jones M, Morgan MY, Wong R (2012). Non-invasive diagnostic assessment tools for the detection of liver fibrosis in patients with suspected alcohol-related liver disease: a systematic review and economic evaluation. Health Technol Assess.

[27] Sandrin L, Fourquet B, Hasquenoph JM, Yon S, Fournier C, Mal F, Christidis C, Ziol M, Poulet B, Kazemi F, Beaugrand M, Palau R (2003). Transient elastography: a new noninvasive method for assessment of hepatic fibrosis. Ultrasound Med Biol.

[28] Mottin CC, Moretto M, Padoin AV, Swarowsky AM, Toneto MG, Glock L, Repetto G (2004). The role of ultrasound in the diagnosis of hepatic steatosis in morbidly obese patients. Obes Surg.

[29] Kuhn JP (2014). Quantitative chemical shift-encoded MRI is an accurate method to quantify hepatic steatosis. J Magn Reson Imaging.

[30] Reeder SB, Cruite I, Hamilton G, Sirlin CB (2011). Quantitative assessment of liver fat with magnetic resonance imaging and spectroscopy. J Magn Reson Imaging.

[31] Heath ML, Kow L, Slavotinek JP, Valentine R, Toouli J, Thompson CH (2009). Abdominal adiposity and liver fat content 3 and 12 months after gastric banding surgery. Metabolism.

[32] Gaborit B et al. Ectopic fat storage in the pancreas using H-MRS: importance of diabetic status and modulation with bariatric surgery-induced weight loss. Int J Obes. 2014; 10.1038/ijo.2014.126 25042860

[33] Verras CG (2017). Serum fetuin-A levels are associated with serum triglycerides before and 6 months after bariatric surgery. Hormones (Athens).

[34] Brix JM, Stingl H, Höllerl F, Schernthaner GH, Kopp HP, Schernthaner G (2010). Elevated Fetuin-A concentrations in morbid obesity decrease after dramatic weight loss. J Clin Endocrinol Metab.

[35] Yang PJ, Ser KH, Lin MT, Nien HC, Chen CN, Yang WS, Lee WJ (2015). Diabetes associated markers after bariatric surgery: Fetuin-A, but not matrix Metalloproteinase-7, is reduced. Obes Surg.

