# Supplementary material for: Quantification of Liver, Subcutaneous, and Visceral Adipose Tissues by MRI Before and After Bariatric Surgery
Source: Obes Surg. 2019 May 14;29(9):2795–805. doi: 10.1007/s11695-019-03897-2 (PMC6713693; doi:10.1007/s11695-019-03897-2)
Supplement: Supplementary file 1 — (DOCX 48 kb) [file 11695_2019_3897_MOESM1_ESM.docx]

**SUPPLEMENTARY INFORMATION**

**Supplementary Materials and Methods**

**Study protocol**

Each subject gave written informed consent for the study. Morbidly obese subjects were recruited by Prof. Dr. Ralph Peterli at St. Claraspital Basel, and lean controls were recruited by word of mouth at the University Hospital of Basel. All patients received a detailed clinical work-up described previously [[1](#_ENREF_1)]. The screening procedure for the present study included the following assessments: a medical interview and a full physical examination. Following the routine of our hospital, the patients did not have to maintain any specific diet (e.g. caloric restriction to decrease liver volume) prior to surgery. Inclusion criteria were: males and females, body-mass index of ≥35 kg/m^2^ (morbidly obese group) resp. >18 and <30 kg/m^2^ (lean controls), age 18-60 years. The morbidly obese patients were scheduled for gastric bypass or sleeve gastrectomy. Exclusion criteria were: smoking, substance abuse, diabetes, history of gastrointestinal disorders, pace-maker, claustrophobia.

**Blood analysis**

Morbidly obese patients and lean controls fasting blood samples were taken on ice into tubes containing EDTA (6 μmol/L). Tubes were centrifuged at 4º C at 3000 rpm for 10 min and plasma samples were stored at -70º C until analysis of fasting glucose, insulin, L-FABP, Fetuin A, M30. Plasma glucose concentration was measured by a glucose oxidase method (Rothen Medizinische Laboratorien AG, Basel, Switzerland). For analysis of insulin, L-FABP, M30 and Fetuin A we used commercially available ELISA kits: insulin (Millipore, St. Charles, Missouri, USA) L-FABP (Hycult biotech, Uden, the Netherlands), Fetuin A (Epitope Diagnostics, Inc., San Diego, U.S.A) and M30 (M30 Apoptosense, Peviva, VLVbio, Nacka, Sweden).

In morbidly obese patients blood lipids (total cholesterol, HDL, LDL and triglycerides) were examined at pre-, and after 6 and 12 months after bariatric surgery. Fasting samples were taken.

**Data analysis, sample size estimation, statistical analysis**

Descriptive statistics were used for demographic variables, such as age, weight, height and BMI. Student’s unpaired t-test was used to test for significant differences in liver biomarkers between lean subjects and obese participants. Repeated measurements ANOVA with Bonferroni correction was used to compare blood parameters (liver biomarkers, insulin, glucose and blood lipids) before vs. after surgery (0-3-6-12 months). Linear regression models were applied to describe associations of %-LF, VAT and SAT and changes in blood parameters at multiple time points (0-3-6-12-24 months). Results are presented as regression coefficient with corresponding SEM and p-values. In order to model the time course of HOMA and fat values, exponential models were selected. Additionally, a linear ascending process was superposed, because a trend of slowly increasing values can be visually observed. The explicit formula is:

*exp(A + B * Time) + A1 * Time*

A= log(initial value), B= decay constant, A1= slope of ascending process

Models were estimated using nonlinear mixed-effects models. Additionally residual plots were displayed to check the goodness of fit (not shown). Regression results are presented as regression coefficients (A, B, A1) with corresponding standard error and p-value. Reproducibility of fat values before surgery was estimated by Intra Class Correlation (ICC) and Coefficient of Variation (CV). ICC is able to quantify the reproducibility of measurements made by different visits measuring the same quantity. The ICC ranges between zero and one, where means perfect agreement. A value between 0.75 and 1.00 can be marked as excellent. Exponential decay constants were compared between all four fat parameters (%-LF, TLV, VAT and SAT) using paired Wilcoxon-Tests. Decay constants were estimated for each subject from the corresponding nonlinear mixed-effects model. Additionally, pairwise correlations between fat parameters were estimated exploratory using spearman correlation. P-values <0.05 were considered as significant. Evaluations were done using R (version 3.3.2) a software for statistical computing and SPSS for Windows, Version 22.0 (SPSS Inc., Chicago, USA) [[2](#_ENREF_2)].

**Supplementary Results**

**Plasma L-FABP, Fetuin A and M30**

In nine morbidly obese patients and 12 healthy lean controls liver biomarkers were measured.

L-FABP

L-FABPs are cytoplasmic proteins, which play an important role in the intracellular utilization of fatty acids, transport and metabolism. In case of liver damage, L-FABP leaks rapidly out of necrotic cells leading to a rise in serum levels and therefore represents a sensitive marker. Before surgery, mean plasma L-FABP was 26.4±8.0 ng/mL and decreased after surgery (at 3 months: 20.6±5.3 ng/mL and at 6 months: 10.2±3.1 ng/mL). However, this was not statistically significant (pre vs. 3 months: p=0.907; pre vs. 6 months: p=0.162, and 3 vs. 6 months: p=0.050). In lean controls, L-FABP concentration was 9.1±2.3 ng/mL. Comparing plasma L-FABP values of morbidly obese patients (before surgery) to plasma L-FABP values of lean controls, we found statistically significant higher values in morbidly obese patients (obese, before surgery vs. lean; p=0.026). Six months after surgery, no statistically significant difference was seen between postoperative patients and lean controls (at 3 months vs. lean; p=0.038 and at 6 months vs. lean; p=0.789); *Supplementary* Figure 2.

Fetuin A

Fetuin A is a glycoprotein, which is synthesized by the liver and secreted into the blood stream. Fetuin A concentrations in the blood are significantly elevated in patients with biopsy-proven NAFLD [[3](#_ENREF_3)]. Before surgery, mean plasma Fetuin A concentration was 0.715±0.062 g/L and decreased minimally after surgery (at 3 months: 0.653±0.063 g/ and at 6 months: 0.662 ±0.052 g/L). However, this was not statistically significant (pre vs. 3 months, pre vs. 6 months and 3 vs. 6 months, p=1.0, respectively). In lean controls, Fetuin A concentration was 0.788±0.097g/L. Comparing plasma Fetuin A values of morbidly obese patients (before surgery) to plasma Fetuin A values of lean controls, we found no statistically significant difference (obese, before surgery vs. lean; p=0.579). No statistically significant differences was seen between postoperative patients and lean controls (at 3 months vs. lean: p=0.309 and at 6 months vs. lean: p=0.328); *Supplementary* Figure 2.

M30

The monoclonal antibody M30 is a specific marker for apoptosis: early in apoptosis, cytokeratin 18 is cleaved by caspases, resulting in exposure of an epitope, which then becomes detectable with M30 [[4](#_ENREF_4)]. In blood samples of various liver diseases elevated cytokeratin 18 fragments are found [[5](#_ENREF_5), [6](#_ENREF_6)]. Before surgery, mean M30 concentration was 162.8±62.1 U/L and decreased after surgery (at 3 months: 118.9±23.2 U/L and at 6 months: 88.1±9.5 U/L). However, this was not statistically significant (pre vs. 3 months p=0.936; pre vs. 6 months p=0.609 and 3 vs. 6 months, p=0.276). In lean controls, M30 concentration was 83.4±3.6 U/L. Comparing M30 values of morbidly obese patients (before surgery) to M30 values of lean controls, we found no statistically significant difference (obese, before surgery vs. lean; p=0.238). No statistically significant differences was seen between postoperative patients and lean controls (at 3 months vs. lean; p = 0.168 and at 6 months vs. lean; p=0.616); *Supplementary* Figure 2.

**Supplementary References**

1. Schneider, R., et al., *The impact of preoperative investigations on the management of bariatric patients; results of a cohort of more than 1200 cases.* Surg Obes Relat Dis, 2018. **14**(5): p. 693-699.

2. Team, R.C., *R: A language and environment for statistical computing. R Foundation for Statistical Computing, Vienna, Austria.* <https://www.R-project.org/>, 2016.

3. Haukeland, J.W., et al., *Fetuin A in nonalcoholic fatty liver disease: in vivo and in vitro studies.* Eur J Endocrinol, 2012. **166**(3): p. 503-10.

4. Leers, M.P., et al., *Immunocytochemical detection and mapping of a cytokeratin 18 neo-epitope exposed during early apoptosis.* J Pathol, 1999. **187**(5): p. 567-72.

5. Wieckowska, A., et al., *In vivo assessment of liver cell apoptosis as a novel biomarker of disease severity in nonalcoholic fatty liver disease.* Hepatology, 2006. **44**(1): p. 27-33.

6. Woolbright, B.L., et al., *Cell Death and Prognosis of Mortality in Alcoholic Hepatitis Patients Using Plasma Keratin-18.* Gene Expr, 2017. **17**(4): p. 301-312.

**Supplementary Figure Legends**

**Supplementary Figure 2: Liver biomarkers**

L-FABP (liver fatty acid-binding protein), Fetuin A and M30. Morbidly obese patients (n=9) before and 3 and 6 months after surgery and healthy lean controls (n=12).
