# Supplementary figures and images for: Quantification of Liver, Subcutaneous, and Visceral Adipose Tissues by MRI Before and After Bariatric Surgery
Source: Obes Surg. 2019 May 14;29(9):2795–805. doi: 10.1007/s11695-019-03897-2 (PMC6713693; doi:10.1007/s11695-019-03897-2)

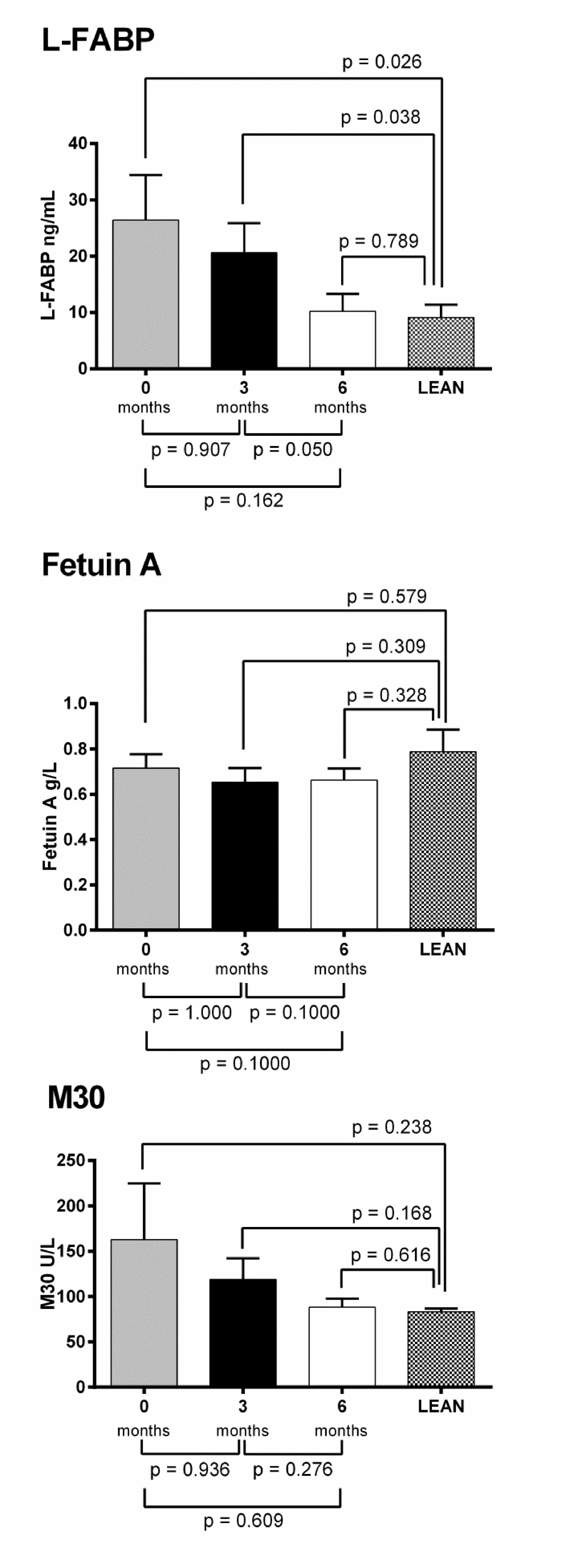

Supplement: Supplementary file 2 — (PNG 201 kb) [file 11695_2019_3897_Fig2_ESM.png]
